# Thoracic Spinal Intradural Arachnoid Cyst With a Fulminant Course

**DOI:** 10.31486/toj.23.0029

**Published:** 2023

**Authors:** Temitayo O. Ayantayo, Oluwafemi F. Owagbemi, Serge Rasskazoff, Olawale A. R. Sulaiman

**Affiliations:** Department of Neurosurgery, RNZ Neurosciences, Victoria Island, Lagos, Nigeria

**Keywords:** *Arachnoid cysts*, *laminectomy*, *paraplegia*, *spinal cord compression*, *spinal intradural arachnoid cysts*, *thoracic vertebrae*

## Abstract

**Background:** Spinal arachnoid cysts are rarely occurring benign cerebrospinal fluid–containing lesions that can occur anywhere along the spinal axis but are principally seen in the thoracic spine. They occur either ventrally or dorsally and can be extradural, intradural extramedullary, or intramedullary. They may be asymptomatic or can present with insidious pain and neurologic symptoms related to spinal cord and/or nerve root compression.

**Case Report:** A 49-year-old male developed sudden midback pain with rapid progression to gait instability, urinary retention, and paraplegia within 10 hours. His presentation for neurosurgical care was delayed because of lack of funds and unfavorable insurance policies. At presentation 3 months after the onset of symptoms, magnetic resonance imaging of his spine showed a ventrolateral intradural extramedullary cystic lesion at T5-T6 with severe cord compression. He underwent T5-T6 and T6-T7 laminectomies with a limited left sixth rib costotransversectomy for microsurgical resection of the cyst. Postoperatively, the patient reported improvement in bladder and bowel control, but his paraplegia persisted.

**Conclusion:** Arachnoid cysts are mostly benign lesions; however, they may have disastrous outcomes if not promptly addressed with the necessary urgency when symptoms are progressive, as in our patient.

## INTRODUCTION

Arachnoid cysts are benign nonneoplastic thin-walled mass lesions in the cranium or spine that contain cerebrospinal fluid (CSF). When located in the spine, arachnoid cysts are classified as extradural, intradural extramedullary, or intramedullary based on their relationship with the dura and spinal cord, and they may arise in the dorsal or ventral part of the spinal canal.^1-3^ In the Nabors et al classification of spinal meningeal cysts, intradural arachnoid cysts are Type III.^4^ Spinal arachnoid cysts are generally less common than their cranial counterparts.^5^ Cranial arachnoid cysts are more common in children than in adults,^6^ while spinal arachnoid cysts are seen more commonly in adults.^7-9^ Arachnoid cysts may have an infective, traumatic, or iatrogenic etiology but can also be idiopathic.^1,10^

Spinal arachnoid cysts are more commonly located in the thoracic region and are mostly dorsal.^2,3,8,11-13^ The majority of spinal arachnoid cysts are asymptomatic; however, some present with clinical features of spinal cord and/or nerve root compression within the spinal canal and intracranial hypotension as a result of CSF drainage into the cyst.^1,10,13^ The natural history of spinal arachnoid cysts is initial asymptomatic growth after which patients develop clinical symptoms. Pain from spinal cord and/or nerve root compression is usually insidious, as are the associated neurologic deficits. The pain most likely arises from cyst enlargement and the resultant stretching of the dura, which receives innervation from the sinuvertebral nerves and the nerve plexuses of the posterior longitudinal ligament and segmental artery radicular branches.^14^ The usual course of the disease is relatively benign, and if intervention occurs early, the prognosis is good.

Magnetic resonance imaging (MRI) is the gold standard for diagnostic imaging to localize the cyst, demonstrate the neural element compression, and differentiate the cyst from other lesions, such as subarachnoid webs and other spinal cysts.^1,8,10^ Syringomyelia may occur in association with arachnoid cysts, presumably because of an alteration in CSF flow dynamics, based on one of the theories for syringomyelia formation.^15^ Treatment of spinal arachnoid cysts is surgical and aimed at decompressing the neural elements and preventing cyst refilling.^1,5,10,11,13,16,17^

We present a case of idiopathic ventrolateral thoracic intradural extramedullary arachnoid cyst with an unusual presentation and review the literature about idiopathic intradural thoracic spinal arachnoid cysts.

## CASE REPORT

A 49-year-old male developed sudden midback pain radiating to the anterior chest wall and was admitted to a hospital in Lagos, Nigeria, a lower-middle-income African country.^18^ The patient developed gait instability within 7 hours of the onset of symptoms and developed urinary retention followed by paraplegia 10 hours after the back pain started. He had associated loss of pain and temperature sensation below the umbilicus and spontaneous bilateral lower limb tonic spasms. The patient had no history of trauma, spinal surgery, infection, or spinal anesthesia. Neuroimaging evaluation was not done at the admitting hospital because the patient did not have the necessary funds. His symptoms progressed to bladder and bowel incontinence during the next 2 months.

**Figure 1.**
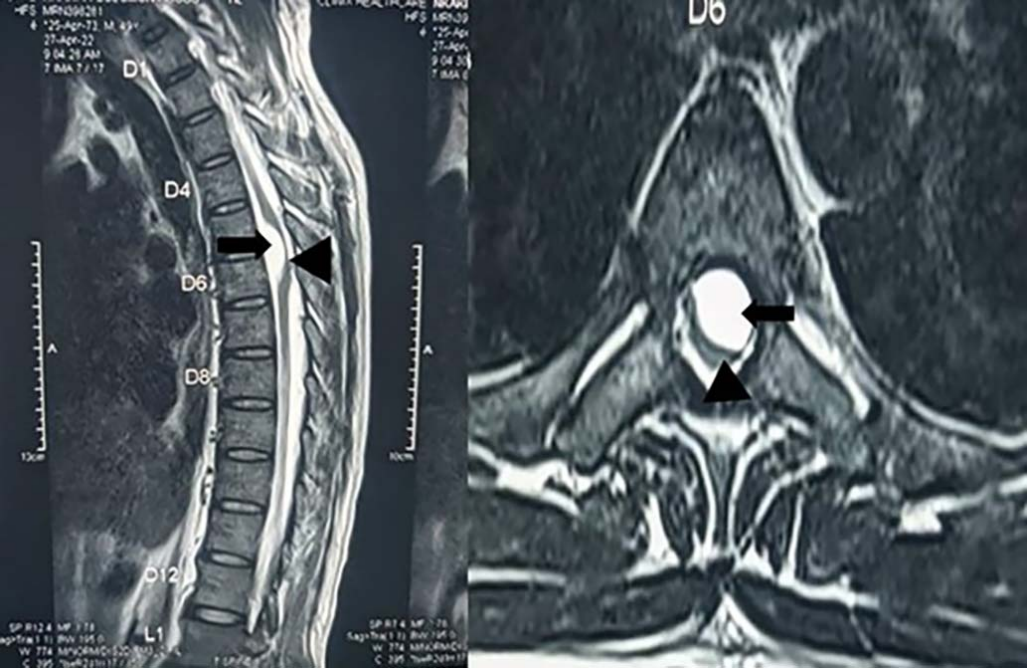
Thoracic spine magnetic resonance imaging sagittal (left) and axial (right) views show the ventral cystic lesion with severe cord compression. Black arrows indicate the arachnoid cyst, and the black arrowheads indicate the compressed and displaced spinal cord.

The patient's presentation to our facility for neurosurgical assessment and care was delayed by his inability to obtain appropriate imaging because of insurance roadblocks and financial challenges until 3 months after the onset of symptoms. Neurologic examination revealed lower extremity power of 0/5, reduced deep tendon reflexes, and extensor plantar responses. He had a T6 sensory level (light touch). MRI of the spine showed a ventrolateral intradural extramedullary cystic lesion spanning T5-T6. The cyst occupied >50% of the cross-section of the spinal canal (Figure 1). The patient was diagnosed with a thoracic spinal arachnoid cyst with American Spinal Injury Association Impairment Scale grade A classification. He was offered surgical resection of the cyst with the goal of improving his chances of recovery from the spinal cord injury.

**Figure 2.**
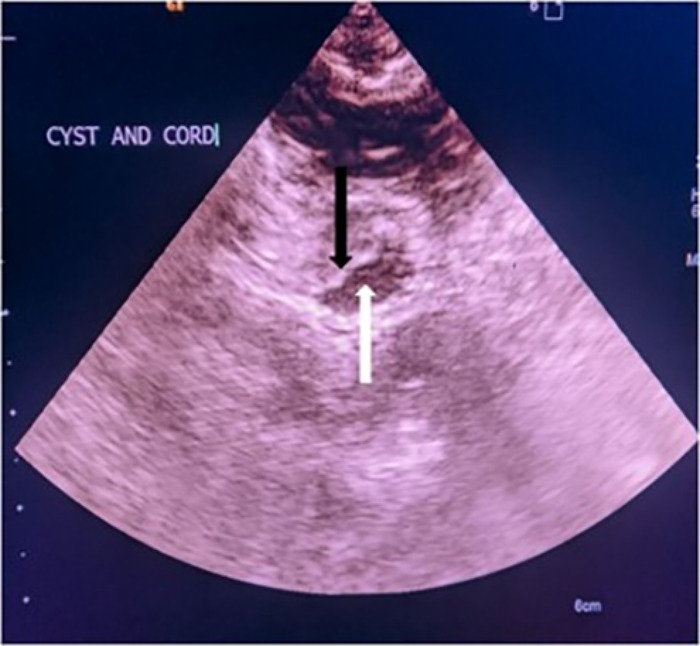
Intraoperative sonogram shows the spinal cord and arachnoid cyst in cross-section. The black arrow indicates the spinal cord, and the white arrow indicates the arachnoid cyst.

**Figure 3.**
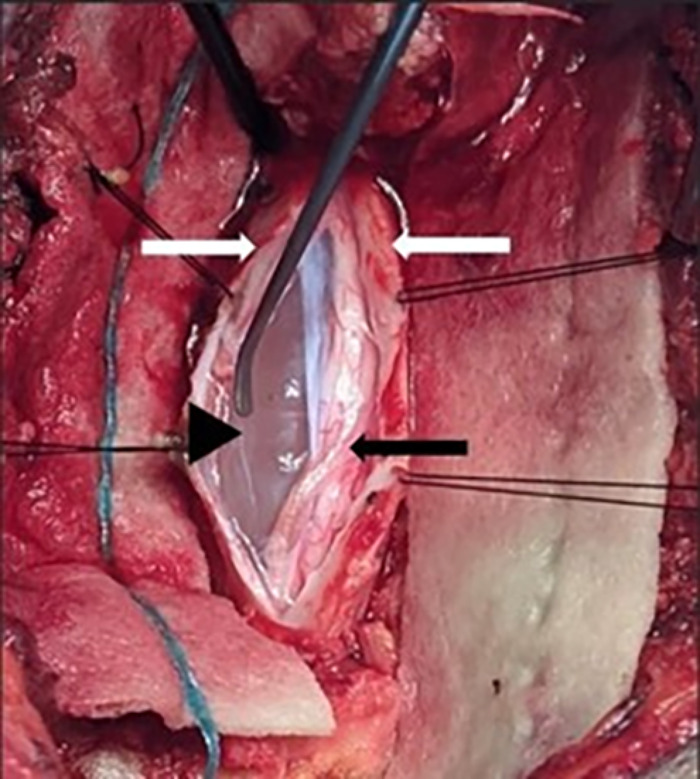
Intraoperative view (via a laminectomy) of the thoracic intradural extramedullary arachnoid cyst. The white arrows indicate retracted edges of the incised dura, the black arrowhead indicates the arachnoid cyst, and the black arrow indicates the compressed spinal cord.

With the aid of fluoroscopic localization, T5-T6 and T6-T7 laminectomies with a limited left sixth rib costotransversectomy were performed, and the craniocaudal extent of the cyst was confirmed with the aid of intraoperative ultrasonography (Figure 2). The fifth and sixth left intercostal nerves were divided to facilitate dura mobilization, and the dura was opened along the line of the dura sleeves. The intradural extramedullary arachnoid cyst was identified and excised using microsurgical techniques (Figure 3). After the decompression, the spinal cord reexpanded intraoperatively.

**Figure 4.**
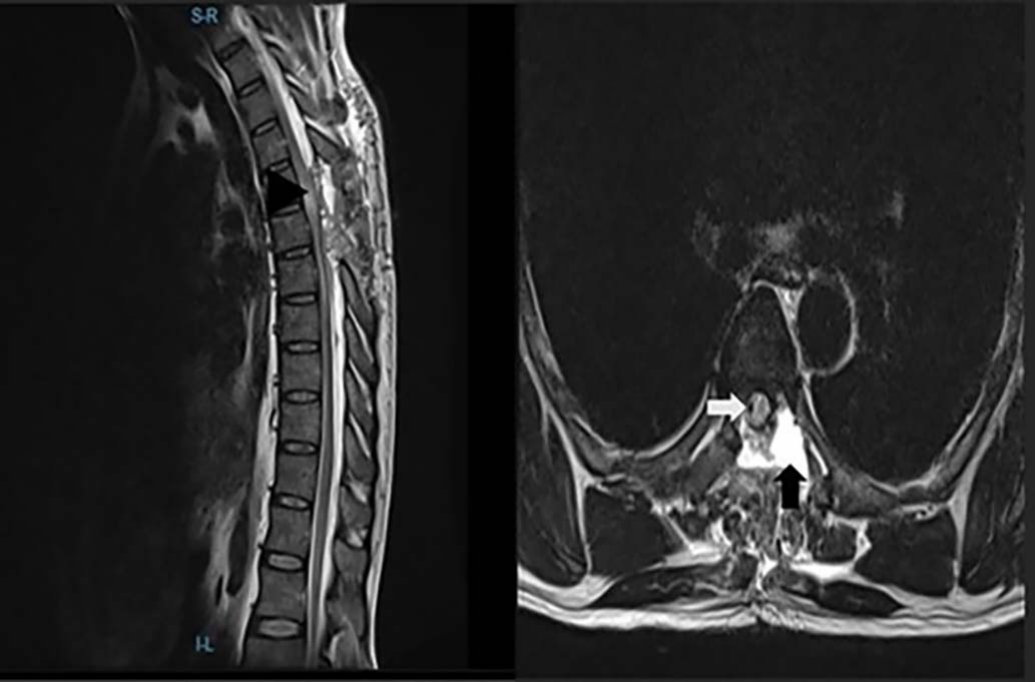
Thoracic spine magnetic resonance imaging sagittal (left) and axial (right) views show complete resection of the arachnoid cyst, cord reexpansion (white arrow), a small extradural cerebrospinal fluid collection (black arrow), and associated cord edema (black arrowhead).

The patient had no complications, and his postoperative MRI showed complete resection of the arachnoid cyst with cord reexpansion to fill the spinal canal in cross-section, associated cord edema, and a small extradural CSF collection (Figure 4).

The patient was discharged to an inpatient rehabilitation facility 4 days after surgery. Histopathologic examination of the resected cyst wall revealed delicate fibrous connective tissue lined by meningothelial cells, confirming the diagnosis of an arachnoid cyst. Three months after surgery, the patient reported improved but not full bladder and bowel control, although he was still paraplegic.

## DISCUSSION

Arachnoid cysts are not common, and idiopathic spinal arachnoid cysts are rare.^10^ Our patient had no apparent cause for the arachnoid cyst, hence our classification of the cyst as idiopathic. Furthermore, intradural arachnoid cysts, which our patient had, are less common than their extradural counterparts.^1,8,9^ Not surprisingly, the cyst in our patient was in the thoracic region, the most common location for arachnoid cysts.^2,11-13^ The ventral location of the cyst was unusual, however, as most spinal arachnoid cysts are dorsally located.^2,3,13^ The thoracic canal is the narrowest in the spinal column, so a space-occupying lesion at that level is expected to have more serious effects and poorer outcomes compared to cysts in the cervical, lumbar, and sacral spinal regions where the canal is more capacious. The relatively shorter duration of symptoms in thoracic arachnoid cysts compared to arachnoid cysts in other spinal regions has been attributed to the narrow canal diameter in that region.^4^ In our patient, symptom progression was rapid—onset of pain to paraplegia within 10 hours.

Spinal arachnoid cysts present with pain, weakness, sensory disturbances, gait abnormalities, and sphincter problems.^2,8,10,13,19^ Pain from spinal arachnoid cysts is usually insidious and chronic rather than sudden, as seen in our patient. Weakness is likewise often insidious, unlike in our patient, in whom it surprisingly progressed rapidly. We believe that the case of this patient is the first report of a fulminant course of this benign pathology, which, if left surgically untreated, will progress to the end of the course—spinal cord injury and disability.

CSF in arachnoid cysts generally flows between the cysts and the subarachnoid space via a connecting opening in the membrane of the cyst, and not all patients will need intervention.^20,21^ In a case report of an extradural cyst by Ergun et al, the only symptom was chronic intermittent low back pain; the patient had no neurologic impairment or cyst enlargement, and the pain improved during a 2-year follow-up.^21^ Cyst growth results from a net inflow, and when the communication site has a 1-way valve effect,^22^ the cyst enlarges rapidly without emptying.

We postulate that sudden pain in a patient with a diagnosed spinal arachnoid cyst who is neurologically intact is a sign of rapid cyst expansion and heralds neurologic deterioration. Such pain should prompt urgent imaging and intervention to prevent the impending rapid neurologic decline. Even if repeat imaging is not immediately available, not delaying surgical decompression may be worthwhile.

We conducted PubMed and Google Scholar searches with the keywords “arachnoid cyst,” “idiopathic,” “thoracic,” and “intradural” and screened the identified articles and cases against our inclusion and exclusion criteria. We included articles about idiopathic and congenital intradural cysts of the thoracic spine and excluded articles about extradural cysts; cysts in the cervical, lumbar, and sacral regions of the spine; and cysts attributable to other causes. From the relevant articles, we tabulated patient demographics, symptoms, duration of symptoms until diagnosis, location of the cyst, affected spine levels, presence of syrinx, treatment offered, and complications following interventions (Table 1).^2,3,11,13,16,17,19,23-46^

**Table 1.** Reports of Idiopathic and Congenital Intradural Thoracic Arachnoid Cysts

Study	Age, years/Sex	Symptoms	Duration of Symptoms	Location	Spine Level(s)	No. of Level(s)	Syrinx	Treatment	Complications
Raja et al, 1970^23^	22/M	Back pain, leg weakness	3 yrs	Dorsal	T6	1	N/A	Laminectomy + cyst excision	N/A
Aarabi et al, 1979^24^ (2 patients)	27/F	LE weakness, back pain	N/A	N/A	T9	1	N/A	Laminectomy + cyst resection	N/A
	57/M	LE weakness, dysesthesia, radiating back pain, difficulty voiding	26 yrs	N/A	T5-6	2	N/A		N/A
Clifton et al, 1987^25^	53/M	Unsteady gait, lower limb feeling of tightness	1 wk	Dorsal	T3-6	4	+	Laminectomy + cyst fenestration	N/A
Rabb et al, 1992^26^ (3 patients)	4.5/M	Associated Gardner syndrome	N/A	Dorsal	T1-T3	3	N/A	Fenestration	N/A
	8/F	Recurrent UTI and constipation	N/A	Dorsal	T3-T5	3	N/A	Fenestration	N/A
	3/F	Back pain	N/A	Dorsal	T4-T9	6	N/A	Cyst-subarachnoid space shunt	N/A
Mascalchi et al, 1993^27^	76/M	Bilateral leg numbness, gait difficulty	20 yrs	Dorsal	T7-T10	4	N/A	Laminectomy + cyst excision	N/A
Fujimura et al, 1996^28^	23/F^a^	Progressive lumbago, back pain, and bilateral hypesthesia	2 yrs	Dorsal	T4-T5, T5-T8	2, 4	N/A	T4-T5 laminotomy + T4-T8 hemilaminectomy + arachnoid membranectomy	N/A
Caruso et al, 1999^29^	40/M	Dorsal pain, LE weakness, gait disturbance, and sexual and urinary dysfunction	1 yr	Ventral	T2	1	N/A	Cyst resection	N/A
Willems et al, 2000^30^	50/F	Loss of LE control	11 yrs	Dorsal	T6-T9	4	N/A	T5-T7 laminectomy + partial cyst resection and fenestration	N/A
Abou-Fakhr et al, 2002^31^	41/M^b^	Interscapular pain, paresthesia	3 yrs	Dorsal	T3-T10	8	N/A	T5-T9 laminectomy	Recurrence
Wang et al, 2003^2^ (13 patients)	67/M	Neuropathic pain, lower extremity weakness and numbness, incontinence, and myelopathy	N/A	Dorsal	T5-T6	2	+	Laminectomy, cyst fenestration + resection of cyst wall, and syrinx shunt to subarachnoid space	Two wound infections, increased leg numbness
	70/M			Dorsal	T5-T8	4	+		
	41/F			Dorsal	T4-T9	6	–		
	54/M			Ventral	T3-T4	2	+		
	38/F			Dorsal	T5-T8	4	+		
	55/M			Dorsal	T7-T9	3	–		
	80/F			Dorsal	T5-T6	2	–		
	38/M			Dorsal	T7-T8	2	–		
	20/M			Ventral	T6-T7	2	–		
	35/M			Ventral	T7-T10	4	+		
	47/F			Dorsal	T5-T6	2	–		
	39/M			Dorsal	T3-T7	5	+		
	67/M			Ventral	T7-T8	2	+		
Bassiouni et al, 2004^13^ (7 patients)	42/F	Gait unsteadiness, pain, lower limb weakness	N/A	Dorsal	T1-T4	4	N/A	Laminectomy + cyst resection	N/A
	67/M				T2-T4	3			
	54/F				T3-T6	4			
	60/M				T3-T6	4			
	41/M				T5-T7	3			
	45/F				T6-T8	3			
	46/F				T9-T12	4			
Wenger et al, 2007^17^	55/F	Multifocal pain and dysesthesia, gait ataxia	5 mos	Dorsal	T5-T6	2	N/A	T5-T7 laminectomy + cyst excision	N/A
Holly and Batzdorf, 2007^11^ (7 patients)	81/M	Gait difficulty (7) Arm pain (2) Abnormal lower extremity sensation (6) Bladder incontinence (2)	N/A	Dorsal	T3-T4	2	+	Laminectomy and cyst resection	Wound dehiscence
	50/M				T5	1			
	54/M				T6-T7	2			
	60/M				T2-T3	2			
	42/M				T5-T6	2			
	35/M				T3-T4	2			
	36/M				T6-T7	2			
da Conceição Araújo Filho et al, 2009^16^	28/F	Pain, paresthesia, progressive lower extremity weakness, gait ataxia	10 mos	Dorsal	T1-T12	12	N/A	T3-T6 laminectomies + cyst fenestration to subarachnoid space and cyst wall resection	N/A
Rao et al, 2010^32^	9/M	Progressive paraparesis, gait ataxia (associated meningocele)	2 wks	Dorsal	T1-T5	5	N/A	T1-T4 laminectomy + cyst resection	N/A
Gómez et al, 2011^33^	72/F^b^	Spastic paraparesis, gait instability	6 mos	Dorsal	T1-T7	7	N/A	Cyst fenestration	Recurrence and syrinx formation
Chern et al, 2011^34^	18 mos/F	Paraparesis	N/A	Dorsal	T1-T9	9	N/A	Laminotomy and fenestration	N/A
Bond et al, 2012^35^ (7 patients)	3/F	Back pain Asymptomatic, spasticity, bilateral LE weakness	N/A	Dorsal	T4-T5	2	N/A	Cyst fenestration	N/A
	6.5/M			Ventral	T1-T12	12			
	12/F			Dorsal	T1-T4	4			
	1.5/M			Dorsal	T12	1			
	6/M			Ventral	T4-T7	4			
	4/M			Dorsal	T7-T10	4			
	4/M			Dorsal	T2-T6	5			
Su et al, 2012^36^	8/M	Progressive LE weakness, bladder and bowel incontinence	3 yrs	Dorsal	T5	1	+	T5-T7 laminoplasty + partial cyst resection + fenestration	N/A
Evangelou et al, 2013^37^ (2 patients)	23 mos/M	Delayed motor development, lower limb spasticity, paraparesis, gait difficulties	N/A	Dorsal	T1-T3	3	N/A	Laminectomy + cyst fenestration + cyst wall excision	N/A
	16 mos/F			Dorsal	T2-T4	3			
Miyashita et al, 2015^38^	69/M	Unsteady gait	N/A	Dorsal	T4-T5	2	N/A	Laminectomy + cyst resection	N/A
Alugolu et al, 2016^39^	54/F	Mid-back pain, lower limb weakness, and paresthesia	1 yr	N/A	T9-T11	3	–	T8-T12 laminectomy	Pseudomeningocele
Viswanathan et al, 2017^40^ (12 patients)	66/F	Lower extremity weakness, gait disturbance, paresthesia, urinary incontinence	N/A	N/A	T2-T3	2	+	Laminectomy + cyst fenestration + partial cyst wall excision	Two cases of VTE
	68/F				T3-T4	2	–		
	35/M				T7-T8	2	–		
	62/M				T7-T8	2	+		
	41/M				T1-T6	6	+		
	50/M				T3-T10	8	+		
	47/M				T6-T7	2	+		
	58/M				T8-T9	2	+		
	61/F				T5-T6	2	–		
	57/F				T4-T10	7	+		
	57/M				T5-T6	2	–		
	40/M				T1-T9	9	+		
Moses et al, 2018^41^ (12 patients)	61/F	Weakness, pain, sensory changes, sphincteric dysfunction, and gait changes	Several days to 4+ yrs	Dorsal	T6-T7	2	–	Laminectomy/laminoplasty + cyst fenestration/excision ± spinal fusion	N/A
	61/M				T3	1	–		
	63/M				T4-T5	2	–		
	29/F				T4-T9	6	+		
	52/F				N/A	N/A	–		
	78/M				T2-T6	5	–		
	39/M				T5	1	–		
	61/F				T4	1	–		
	78/M				T4-T5	2	–		
	67/M				T4-T5	2	–		
	19/M^a^				T3-T5, T8-T10	3, 3	–		
	35/M				T6-T7	2	–		
Fam et al, 2018^3^ (5 patients)	38/F	Back pain, weakness, gait ataxia	2 mos	Dorsal	T5-T12	8	N/A	Resection	N/A
	73/F		24 mos	Ventral	T5-T7	3		Observation	
	51/F		3 mos	Dorsal	T2-T7	6		Resection	
	57/F		8 mos	Dorsal	T3-T8	6		Resection	
	62/F		6 mos	Dorsal	T3-T8	6		Observation	
Eroglu et al, 2019^42^ (4 patients)	51/F	Leg pain	N/A	N/A	N/A	N/A	N/A	Laminectomy + cyst fenestration	N/A
	42/M	Leg pain							
	33/F	Back pain							
	36/M	Back pain, sensory loss							
Himes et al, 2019^43^	60/F	Pain between shoulder blades	1 yr	Dorsal	T3-T7	5	N/A	Laminectomy and cyst fenestration	N/A
Watanabe et al, 2019^44^	37/F	Lower abdominal pain + acute paraparesis	2 wks	Dorsal	T7	1	N/A	T6-T8 hemilaminectomy + cyst excision	N/A
Sadek and Nader-Sepahi, 2019^19^ (17 patients)	64/F	Paraesthesia, neuropathic pain, weakness, and gait unsteadiness	7 mos	Dorsal	T6-T7	2	–	Laminectomy + cyst excision Observation in 1 patient	N/A
	70/F		60 mos		T4-T5	2	–		N/A
	49/M		12 mos		T2-T3	2	–		N/A
	63/F^b^		6 mos		T4-T9	6	+		Recurrence
	41/M		12 mos		T3-T5	3	–		N/A
	60/M		60 mos		T2-T3	2	–		N/A
	49/F		24 mos		T7-T9	3	+		N/A
	57/M		32 mos		T5-T6	2	–		N/A
	77/M		15 mos		T8-T11	4	–		N/A
	77/F		12 mos		T3-T6	4	–		N/A
	25/M^b^		8 mos		T9	1	–		Recurrence
	77/F		12 mos		T3-T6	4	–		N/A
	68/M		36 mos		T7	1	+		N/A
	61/M		6 mos		T4	1	+		N/A
	37/M		12 mos		T8-T9	2	+		N/A
	64/M		6 mos		T2-T3	2	–		N/A
	50/M		6 mos		T5-T6	2	–		N/A
Ebot et al, 2020^45^	80/F	Back pain, urinary incontinence, gait difficulty	N/A	Dorsal	T4-T10	7	N/A	T4-T5 and T9-T10 laminectomies and cyst fenestration	N/A
Kawaguchi et al, 2022^46^	28/F	Left LE weakness, gait disturbance	3 yrs	Dorsal	T3-T4	2	N/A	T3-T6 laminectomy + cyst excision	N/A

^a^Patient had >1 cyst.

^b^Patient required repeat surgery because of cyst recurrence.

LE, lower extremity, N/A, not available; UTI, urinary tract infection; VTE, venous thromboembolism.

We identified 110 patients with clearly defined idiopathic and congenital purely thoracic intradural arachnoid cysts. A summary of their data is presented in Table 2. Most of the patients were male (58%), and patients in the seventh decade of life had the highest prevalence. Cysts spanning 2 vertebral levels had the highest occurrence (37%), while 2 cysts extended across 12 vertebrae, and 2 patients had more than 1 cyst (a 19-year-old male reported by Moses et al^41^ and a 23-year-old female reported by Fujimura et al^28^), for a total of 112 cysts. Most of the cysts (76%) were located dorsally, with 7% located ventrally and the location unreported for 17% of the cysts. Associated syringomyelia was reported for 27% of the patients, syrinx association was not reported in 42%, and the remainder of the patients had no syrinx. Almost all patients (97%) underwent surgical intervention, and surgical complications were reported in 10%. Three patients had their cysts observed without surgical intervention. Reported complications included wound dehiscence, surgical site infection, pseudomeningocele, venous thromboembolism, and 4 cases of recurrence, with 1 associated with syrinx. Four patients required a repeat surgery because of incomplete cyst shrinkage/cyst recurrence.

**Table 2.** Summary of Patient Demographics and Disease Findings, n=110

Variable	n (%)
Sex	
Male	64 (58)
Female	46 (42)
Age, years	
0-10	14 (13)
11-20	3 (3)
21-30	7 (6)
31-40	16 (15)
41-50	18 (16)
51-60	20 (18)
61-70	21 (19)
71-80	10 (9)
81-90	1 (1)
Cyst location	
Dorsal	85^a^ (76)
Ventral	8 (7)
Unreported	19 (17)
Number of vertebral levels^b^	
1	13 (12)
2	40 (37)
3	14^c^ (13)
4	17^c^ (16)
5	5 (5)
6	8 (7)
7	3 (3)
8	3 (3)
9	2 (2)
10	0 (0)
11	0 (0)
12	2 (2)
Unreported	5 (5)
Presence of syrinx	
Yes	30 (27)
No	34 (31)
Unreported	46 (42)
Surgical intervention	
Yes	107 (97)
No	3 (3)

^a^Two patients had 2 separate cysts that were located dorsally, giving a total of 112 cysts in 110 patients.

^b^Levels were not reported in 5 patients; percentages are calculated based on a denominator of n=107.

^c^Two patients had 2 separate cysts that spanned >2 vertebral levels.

Because most spinal arachnoid cysts are dorsally located, the most common approaches to their resection are posterior, especially laminectomy.^13^ Because our patient's arachnoid cyst was located ventrally, a laminectomy would not have granted adequate access for cyst resection without further cord injury, so we performed a limited costotransversectomy. Although our localization of the laminectomy levels using fluoroscopy was accurate, ultrasonography, as has been practiced by other researchers,^11^ aided further in guiding the limits of our durotomy.

Observation, cyst resection, marsupialization, fenestration, and cyst shunts are treatment options for spinal arachnoid cysts.^3,5,11^ We believe that microsurgical resection, which we performed on our patient's cyst, gives the best chance of cure.

Early surgical intervention provides the best chance for resolution of symptoms and functional recovery, with the most improvement noted in gait and motor function; improvement in neuropathic pain and numbness is unpredictable.^2,16,19^

Our review of literature showed low rates of varying postoperative complications (10%), including a low recurrence rate. Poor neurologic recovery has been observed in long-standing cases of myelopathy and has been attributed to secondary spinal atrophy or myelomalacia.^13,16,17^ Our patient did not have any postoperative complications.

## CONCLUSION

Arachnoid cysts are benign lesions. However, they can have disastrous consequences if not treated urgently when symptoms are precipitous or progressive. Our patient had such consequences because of delays in accessing appropriate care.

## Notes

*This article meets the Accreditation Council for Graduate Medical Education and the American Board of Medical Specialties Maintenance of Certification competencies for Patient Care and Medical Knowledge.*
